# Complete Genome Sequence of the Plant Growth-Promoting Bacterium Pantoea agglomerans Strain UAEU18, Isolated from Date Palm Rhizosphere Soil in the United Arab Emirates

**DOI:** 10.1128/MRA.00174-20

**Published:** 2020-04-23

**Authors:** AlReem S. Alkaabi, Naganeeswaran Sudalaimuthuasari, Biduth Kundu, Raja S. AlMaskari, Yasmeen Salha, Khaled M. Hazzouri, Khaled A. El-Tarabily, Synan F. AbuQamar, Khaled M. A. Amiri

**Affiliations:** a Department of Biology, College of Science, United Arab Emirates University, Al Ain, United Arab Emirates; b Khalifa Center for Genetic Engineering and Biotechnology, United Arab Emirates University, Al Ain, United Arab Emirates; University of Arizona

## Abstract

We report the complete genome sequence of the plant growth-promoting bacterium Pantoea agglomerans strain UAEU18. Genome assembly of *P. agglomerans* strain UAEU18 resulted in a single gapless circular genome of 4.04 Mb, three associated plasmids (plasmid 1, 513,383 bp; plasmid 2, 86,850 bp; and plasmid 3, 184,488 bp), and a total of 4,556 gene models.

## ANNOUNCEMENT

Plant growth-promoting rhizobacteria (PGPR) are a class of beneficial free-living bacteria inhabiting the soil ecosystem on or near the roots of the plant (i.e., the rhizosphere) (1). PGPR have the potential capability to significantly enhance the yields of various crops. PGPR stimulate the growth of plants by one or more of a number of different direct and indirect mechanisms (2). The PGPR Pantoea agglomerans strain UAEU18 was isolated from rhizosphere soil of date palm trees located in the emirate of Ras Al-Khaimah, United Arab Emirates (25.8007°N, 55.9762°E). In the laboratory, 10 g of the air-dried and sieved rhizosphere soil sample was dispensed into 100 ml of sterile 1 g/liter agar (Sigma-Aldrich Chemie GmbH, Germany) solution in deionized water containing 20 g glass beads (3-mm diameter). The soil suspension was then shaken on a rotary shaker (model G76, New Brunswick Scientific, Edison, NJ, USA) at 350 rpm for 35 min at 28°C. Tenfold dilutions (10^−2^ to 10^−6^) were made in sterile deionized water, and 0.2-ml aliquots were spread with a sterile glass rod over the surface of nutrient agar medium (Lab M Limited, Lancashire, UK) in sterile plastic 9-cm-diameter petri plates. The plates were dried in a laminar flow cabinet for 15 min before incubation at 28°C in the dark. The bacterial colonies were transferred onto nutrient agar plates, and all isolates were stored in 20% glycerol at –80°C.

The PGPR *P. agglomerans* strain UAEU18, obtained from a single colony, was cultivated in nutrient broth at 30°C for 24 h, and the total genomic DNA from the bacterial culture was extracted with an XpressDNA bacterial kit (MagGenome Technologies, Chennai, India) per the protocol instructions. The DNA quality and quantity were confirmed using agarose gel electrophoresis, a NanoDrop 2000 spectrophotometer (ThermoFisher Scientific, Waltham, MA), and a Qubit 2.0 fluorometer (ThermoFisher Scientific).

The initial bacterial strain typing, strain identity, and purity were determined using 16S rRNA gene sequencing. The 16S rRNA gene primers (forward, 5′-AGAGTTTGATCCTGGCTCAG-3′; reverse, 5′-GGTTACCTTGTTACGACTT-3′) were used to amplify the complete 16S rRNA gene, which was sequenced using the Genetic Analyzer 3500 (using both primers). Sequences of the 16S rRNA gene were matched to the NCBI nucleotide bacterial database (online BLAST; 3), and the bacterial species and genus were determined to be Pantoea agglomerans (E value, 0.0).

Genomic DNA was sequenced on an Oxford Nanopore MinION flow cell (FLO-MIN106D R9.4 revision D chip) after preparation using the ligation sequencing kit (SQK-LSK 109). A short-read sequencing library for Illumina sequencing was prepared using a NEBNext Ultra II DNA library preparation kit, and the sequencing was done on an Illumina NovaSeq 6000 sequencer (150-bp paired-end chemistry).

MinION read base calling, demultiplexing, and adapter trimming were performed using Guppy v.3.3.2 (Oxford Nanopore, Cambridge, UK), implemented in the MinKNOW v.3.5.5 interface (Oxford Nanopore). In total, 964,832 long reads were obtained from the MinION sequencer, with 2,244,747,226 bp of nucleotides to generate an estimated ∼450× coverage. The read lengths ranged from 88 to 85,972 bp, with an *N*_50_ value of 3,790 bp. Canu v.1.8 (4) was used for long-read error correction, and corrected reads with a length greater than 1,000 bp were considered for the genome assembly. The Illumina raw data quality was assessed with FastQC (5); adapter and low-quality regions were trimmed using the Trimmomatic v.0.39 program (6). After adapter and low-quality read trimming, 18,119,470 (quality [Q], >30) paired-end reads (read lengths ranged between 50 and 150 bp) were obtained. We applied a hybrid *de novo* genome assembly strategy using SPAdes v.3.11.1 (7) with default settings. Assessment of the quality and completeness of the genome was done using BUSCO v.3 (8). Gene prediction and genome annotation were done using NCBI PGAP (9).

The hybrid assembly resulted in a single gap-free circular genome of 4,040,629 bp, with a GC content of 55.5% and three plasmids (plasmid 1, 513,383 bp; GC content, 53.6%; plasmid 2, 86,850 bp; GC content, 54.2%; plasmid 3, 184,488 bp; GC content, 52.1%). Genome annotation resulted in 4,556 gene models (coding DNA sequences [CDSs], 4,360; rRNAs, 22; tRNAs, 80; noncoding RNAs [ncRNAs], 15; pseudogenes, 79). Preliminary analysis of the annotated genes paves the path to identify the key genes involved in nitrogen metabolism. A detailed functional study of the annotated genes and their corresponding gene expression regulation is in progress.

### Data availability.

The Illumina and Oxford Nanopore reads synthesized during this experiment have been submitted to the NCBI SRA database under BioProject accession number PRJNA602576 and BioSample accession numbers SRR10953135 (Illumina paired-end reads) and SRR10953134 (Oxford Nanopore reads). The assembled genome and plasmid sequences were submitted to NCBI GenBank under accession numbers CP048033 (genome), CP048034 (plasmid 1), CP048035 (plasmid 2), and CP048036 (plasmid 3). The bacterial 16S rRNA gene marker sequenced during this study is available in NCBI GenBank under accession number MN971673.
